# Deep-Sea Origin and In-Situ Diversification of Chrysogorgiid Octocorals

**DOI:** 10.1371/journal.pone.0038357

**Published:** 2012-06-18

**Authors:** Eric Pante, Scott C. France, Arnaud Couloux, Corinne Cruaud, Catherine S. McFadden, Sarah Samadi, Les Watling

**Affiliations:** 1 Department of Biology, University of Louisiana at Lafayette, Lafayette, Louisiana, United States of America; 2 GENOSCOPE, Centre National de Séquençage, Evry, France; 3 Department of Biology, Harvey Mudd College, Claremont, California, United States of America; 4 Département Systématique et Evolution, UMR 7138 UPMC-IRD-MNHN-CNRS (UR IRD 148), Muséum national d’Histoire naturelle, Paris, France; 5 Department of Biology, University of Hawaii at Manoa, Honolulu, Hawaii, United States of America; 6 Darling Marine Center, University of Maine, Walpole, Maine, United States of America; Heriot-Watt University, United Kingdom

## Abstract

The diversity, ubiquity and prevalence in deep waters of the octocoral family Chrysogorgiidae Verrill, 1883 make it noteworthy as a model system to study radiation and diversification in the deep sea. Here we provide the first comprehensive phylogenetic analysis of the Chrysogorgiidae, and compare phylogeny and depth distribution. Phylogenetic relationships among 10 of 14 currently-described Chrysogorgiidae genera were inferred based on mitochondrial (*mtMutS*, *cox1*) and nuclear (18S) markers. Bathymetric distribution was estimated from multiple sources, including museum records, a literature review, and our own sampling records (985 stations, 2345 specimens). Genetic analyses suggest that the Chrysogorgiidae as currently described is a polyphyletic family. Shallow-water genera, and two of eight deep-water genera, appear more closely related to other octocoral families than to the remainder of the monophyletic, deep-water chrysogorgiid genera. Monophyletic chrysogorgiids are composed of strictly (*Iridogorgia* Verrill, 1883, *Metallogorgia* Versluys, 1902, *Radicipes* Stearns, 1883, *Pseudochrysogorgia* Pante & France, 2010) and predominantly (*Chrysogorgia* Duchassaing & Michelotti, 1864) deep-sea genera that diversified *in situ*. This group is sister to gold corals (Primnoidae Milne Edwards, 1857) and deep-sea bamboo corals (Keratoisidinae Gray, 1870), whose diversity also peaks in the deep sea. Nine species of *Chrysogorgia* that were described from depths shallower than 200 m, and *mtMutS* haplotypes sequenced from specimens sampled as shallow as 101 m, suggest a shallow-water emergence of some *Chrysogorgia* species.

## Introduction

Corals of the family Chrysogorgiidae Verrill, 1883 are conspicuous members of deep benthic assemblages. They are found in all major oceans, as far north as Iceland [1] and as far south as Antarctica [2]. They have been described from a variety of habitats, including shallow-water reefs [3], soft sediments, and hard bottoms (e.g., [4]). They were recently described as predominant members of benthic communities on NW Atlantic seamounts [5], [6]. The Chrysogorgiidae are particularly diverse, with about one hundred described species. In his original description of the family, Verrill [7] presented the Chrysogorgiidae as including “some of the most beautiful and interesting of all the known Gorgonians.”

The family ranges between approximately 100 and 3375 m depth [8], most species (>75%) inhabiting deep waters. The family is an assemblage of deep-water specialists (e.g., *Metallogorgia* Versluys, 1902 and *Iridogorgia* Verrill, 1883), a shallow-water specialist (*Stephanogorgia* Bayer & Muzik 1976), and eurybathic genera (*Chrysogorgia* Duchassaing & Michelotti, 1864 and *Radicipes* Stearns, 1883). The variety and gradualism in the bathymetric range of the family makes it a noteworthy model system for the study of diversification and radiation in the deep sea. However, these advantages as a model system have little value if the family Chrysogorgiidae is an artificial assemblage of polyphyletic taxa.

Indeed, despite their ubiquity and relative abundance, little is known about the phylogeny of chrysogorgiid corals. The evolutionary history of the Chrysogorgiidae has, to date, not been appropriately studied. McFadden et al. [9], in their genus-level phylogenetic reconstruction of the subclass Octocorallia Haeckel, 1866, included four of the 12 genera described at the time, and retrieved a monophyletic group. However, the specimens used were all from deep waters and did not cover the morphological, ecological and biogeographic variation observed in the family.

In fact, most octocoral families – as they are currently known – are likely not monophyletic. McFadden et al. [9] included 28 of the 44 octocoral families in their analysis, 14 of which were represented by multiple genera. Only five of these 14 were monophyletic, but even these were based on limited taxonomic data: only a third or less of the genera described in each of these five families were analyzed. Under-representation was particularly important for the Isididae Lamouroux, 1812, for which only two out of 38 described genera (5%) were included, and these two both from only 1 of the 4 subfamilies.

In light of the prevalence of polyphyly among currently-described octocoral families, there is little guarantee that a phylogenetic analysis of the Chrysogorgiidae based on broader taxonomic sampling will recover a monophyletic group. In addition, none of the molecular phylogenies including chrysogorgiids produced to date [9]–[14] have assessed the monophyly of genera. The genus *Chrysogorgia*, in particular, is the most speciose (60+ species) and geographically the most wide-ranging of the Chrysogorgiidae [8]. As *Chrysogorgia* makes about 60% of the species diversity in the family, assessing its monophyly is of particular importance.

Our first goal was to test the monophyly of the family Chrysogorgiidae. Second, we compared depth distributions and evolutionary relationships among genera to evaluate the hypothesis that the two are correlated (i.e., shallow-water taxa are derived from deep-water ones, or vice-versa). We inferred phylogenetic relationships based on taxa from 10 of 14 currently-recognized genera, using mitochondrial (*mtMutS* and *cox1*) and nuclear (18S) markers. Bathymetric ranges were estimated based on our collections, museum records, and a literature review. The family Chrysogorgiidae is put in a broad evolutionary context by the inclusion of DNA sequences and distributional information from all other families of the suborder Calcaxonia (the Isididae, Primnoidae Milne Edwards, 1857, Ellisellidae Gray, 1859, and Ifalukellidae Bayer, 1955).

## Results

### Informativeness and Congruence among Genetic Markers

The information contents of *mtMutS*, *cox1* and 18S were assessed by looking at 42 calcaxonian colonies (Table 1). The 5′ end of *mtMutS* was the most variable region, with 31% of sites being variable. The gene as a whole is slightly less variable (28%). 18S and *cox1*1 were 50% less variable than *mtMutS* (Table 1). The same pattern was observed for parsimony-informative sites. A total of 34 haplotypes were differentiated by the first 781 bp of the *mtMutS* alignment. The entire gene sequence differentiated 37 haplotypes, while *cox1* and 18S differentiated 28 and 40 haplotypes, respectively. All *cox1* haplotypes were also differentiated by *mtMutS*. The additional richness observed at 18S is attributed to ambiguous positions. The molecular variation found at *mtMutS* and *cox1*, and the diagnostic potential of these markers for barcoding is further detailed in [15].

**Table 1:** Length and information content of *mtMutS*, *cox1* and 18S alignments, alone and concatenated.

	Alignment	N. taxa	N. nt	Nt. min-max	N. var	N. pars	Model (AIC)	Model (BIC)
1	*mtMutS* 5′	105	829	691–799	384 (46%)	259 (31%)	TVM+G	TVM+G
2	*mtMutS* 5′ (Gblocks)	105	688	679–688	316 (46%)	227 (33%)	TVM+G	TVM+G
3	*mtMutS*	46	3150	2889–2997	896 (28%)	594 (19%)	TVM+I+G	TVM+G
4	*mtMutS* (Gblocks)	46	2871	2832–2871	837 (29%)	571 (20%)	TVM+I+G	TVM+G
5	*cox1*	64	786	786–786	133 (17%)	92 (12%)	TVM+I+G	TPM1uf+G
6	18S	64	1315	1293–1307	215 (16%)	181 (14%)	GTR+I+G	TIM2ef+I+G
7	*mtMutS* (5′), cox1, 18S	64	2924	2773–2880	645 (22%)	478 (16%)	GTR+I+G	GTR+I+G
8	*mtMutS*, cox1, 18S	42	5236	4969–5078	1192 (23%)	819 (16%)	GTR+I+G	TVM+I+G
9	*mtMutS* (5′)	42	781	691–769	245 (31%)	168 (22%)		
10	18S	42	1300	1293–1297	189 (15%)	160 (12%)		
11	*cox1*	42	786	786–786	108 (14%)	72 (9%)		
12	*mtMutS*	42	3150	2889–2997	895 (28%)	587 (19%)		

Alignments 1–8 were used in phylogenetic analyses. Alignments 8–12 were used to compare levels of variation and information content, based on data from 42 individuals. Gblocks: alignment shortened using Gblocks. N. nt: alignment length in nucleotides. Nt. min-max: shortest and longest sequences (de-gapped). N. var and N. pars: number of variable and parsimony-informative sites (and percentage of total alignment length). Model (AIC) and Model (BIC): model of evolution that best described the data, based on jModelTest runs.

**Figure 1:**
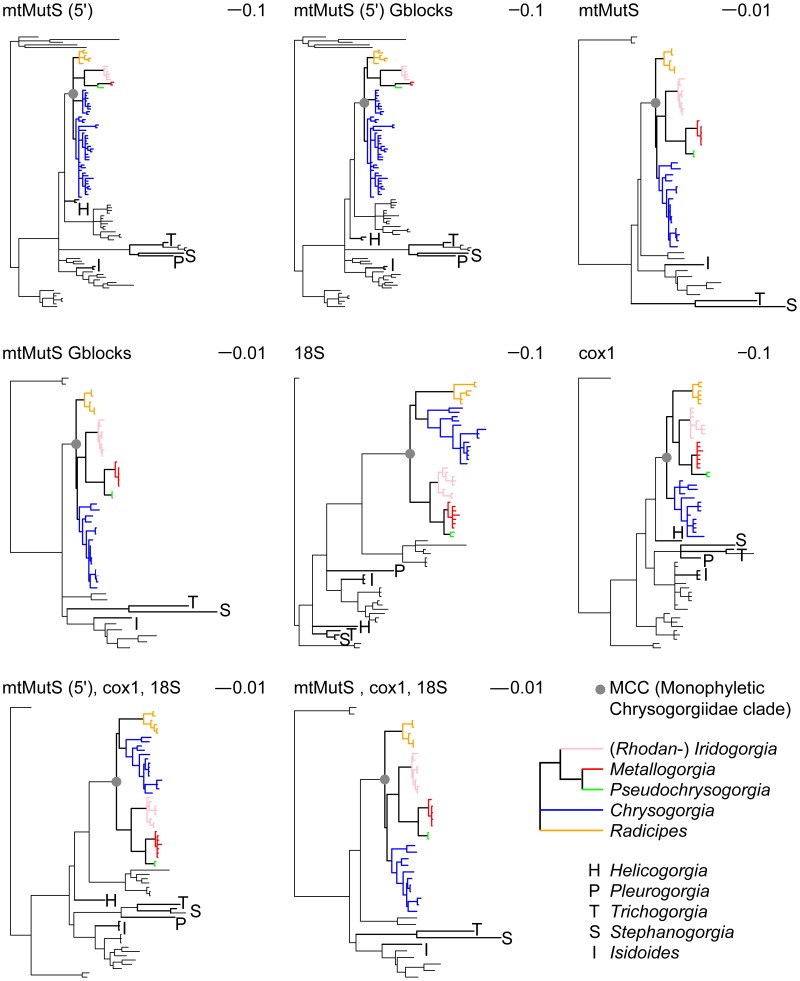
Bayesian 50% majority rule consensus trees based on different markers (*mtMutS*, *cox1* and 18S) and marker combinations. For *mtMutS*, the effect of indels on phylogeny inference was tested by removing them with Gblocks. Chrysogorgiidae taxa are either color coded (MCC) or have bolded branches (nonMCC).The MCC clade is evidenced by a gray circle. All trees are rooted to the Pennatulacea, except trees using the entire *mtMutS* gene (rooted to the Ellisellidae). For sake of clarity, tip labels and node support values were removed, but can be consulted on Figure S1.

The phylogenetic signal contained in the three markers was congruent overall, and recovered the same clades at the genus and family levels (see below and Figure 1). Concatenating markers significantly improved clade support, with the notable exception of the relationship between *Chrysogorgia*, *Radicipes*, and the clade composed of *Iridogorgia*, *Rhodaniridogorgia* Watling, 2007, *Metallogorgia*, and *Pseudochrysogorgia* Pante & France, 2010 (see below). Removing complex indels of doubtful homology at *mtMutS* did not significantly improve taxonomic resolution. Indel removal, however, had the effect of influencing branch lengths (Figures 1 and S1).

### Family Monophyly

**Figure 2:**
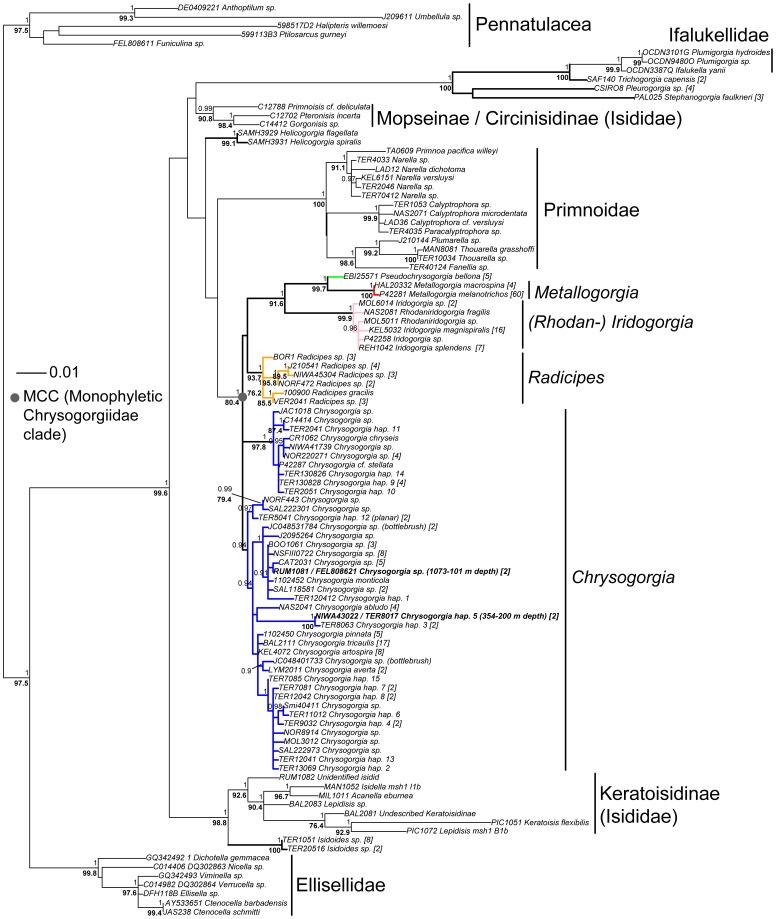
Maximum likelihood reconstruction of the suborder Calcaxonia (rooted to five sea pens; Order Pennatulacea) based on the 5′ end of *mtMutS* (102 taxa, 272 colonies, 829 bp; TVM+G model; 1000 bootstrap replicates). All five families of the Calcaxonia are represented. Node support values from the ML analysis (bold, only values >70% shown) are indicated under each node, and node support values from the Bayesian analysis (>0.90) are above each node. Chrysogorgiidae taxa are either color coded (MCC) or have bolded branches (nonMCC). The collection depth of the shallowest *Chrysogorgia* specimens (≤200 m) for which a DNA sequence could be produced is indicated next to the tip label (bolded).

**Figure 3:**
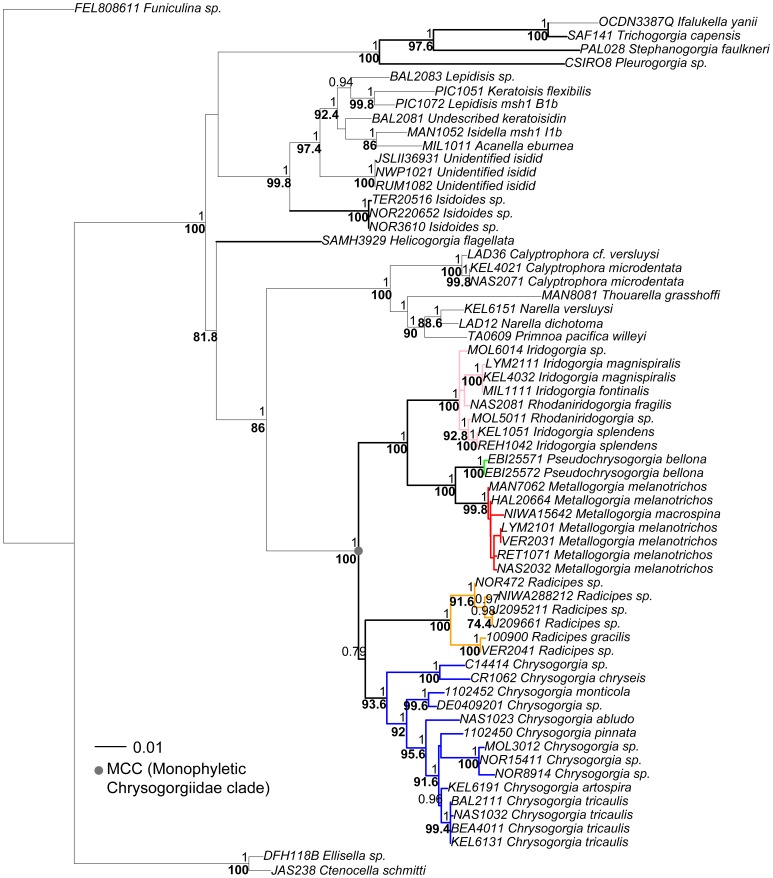
Maximum likelihood reconstruction of the suborder Calcaxonia (rooted to *Funiculina*, a sea pen) based on concatenated sequences of the 5′ end of *mtMutS*, *cox1* and 18S (64 taxa, 2924 bp; GTR+I+G model; 500 bootstrap replicates). All five families of the Calcaxonia are represented. Node support values from the ML analysis (>70%, bold) are indicated under each node, and node support values from the Bayesian analysis (>0.90) are above each node. Chrysogorgiidae taxa are either color coded (MCC) or have bolded branches (nonMCC).

Genetic data strongly suggest that the family as currently described is not monophyletic. The following genera formed a strongly-supported phylogenetic clade: *Iridogorgia*, *Rhodaniridogorgia*, *Metallogorgia*, *Pseudochrysogorgia*, *Radicipes* and *Chrysogorgia* (Figures 2 and 3). We refer to this group as the “monophyletic Chrysogorgiidae clade” (MCC), as it is the only monophyletic group retrieved for genera within the family. In addition, the type species for the family, *Chrysogorgia desbonni* (Duchassaing & Michelotti, 1864), is from a genus in this clade. While the phylogenetic relationship between *Radicipes* and *Chrysogorgia* was difficult to retrieve with strong support, *Iridogorgia*, *Metallogorgia* and *Pseudochrysogorgia* consistently formed a very well-supported monophyletic clade.

All other genera for which we were able to retrieve DNA sequences fell outside of that clade. *Stephanogorgia*, *Pleurogorgia* Versluys, 1902 and *Trichogorgia* Hickson, 1904 formed a strongly-supported clade with the monophyletic ifalukellid genera *Ifalukella* Bayer, 1955 and *Plumigorgia* Nutting, 1910. *Helicogorgia* Bayer, 1981 systematically clustered outside the MCC, either sister to that clade (*cox1* phylogeny), sister to the Primnoidae Milne Edwards, 1857/MCC clade (phylogenies based on *mtMutS* 5′ and concatenation of *mtMutS* 5′, *cox1* and 18S) or sister to the clade composed of *Stephanogorgia*, *Pleurogorgia* and *Trichogorgia* (18S phylogeny). The entire *mtMutS* gene from *Helicogorgia* specimens was not sequenced. The genus *Isidoides* Nutting, 1910 was sister to the monophyletic sub-family Keratoisidinae (Isididae Lamouroux, 1812). This position is conserved across markers and combination of markers with strong statistical support. Only limited genetic data could be extracted from specimens of *Chalcogorgia* Bayer, 1949 (first 187 nt of *mtMutS*). This short sequence grouped with *Helicogorgia* with poor statistical support (56% node support on the ML phylogeny based on the 5′ end of *mtMutS*). The large amount of missing data negatively affected node support throughout the phylogeny; data from *Chalcogorgia* were therefore removed from all other analyses. Despite numerous attempts, no DNA could be amplified from specimens of *Xenogorgia* Bayer & Muzik, 1976. Specimens of *Distichogorgia* Bayer, 1979 could not be obtained.

### Genus Monophyly

Within the MCC, *Metallogorgia*, *Pseudochrysogorgia* and *Radicipes* always formed monophyletic clades with strong statistical support. While *Chrysogorgia* formed two clades in the MCC based on the 5′ end of *mtMutS* (Figure 2), the genus formed a well-supported monophyletic clade based on the multiple-gene phylogeny (Figure 3). Genetic data cannot distinguish the genera *Iridogorgia* and *Rhodaniridogorgia* from each other, and there is very little divergence among haplotypes (uncorrected p distances at the 5′ end of *mtMutS* range from 0.1 to 0.7%; Figure 2 and 3). Outside the MCC, genera *Stephanogorgia*, *Helicogorgia* and *Trichogorgia* (*n* = 3, 5, 5 nominal species, respectively) are represented by multiple specimens (*n* = 3, 2, 2, respectively), but single haplotypes. Genus monophyly could therefore not be assessed. The two haplotypes sampled for the monotypic genus *Isidoides* formed a monophyletic clade.

### Richness and Geographic Distribution of *mtMutS* Haplotypes

*Chrysogorgia* is the richest genus in terms of haplotypic diversity at *mtMutS*: we obtained 41 haplotypes, 31 of which were sampled in the Pacific and 11 from the Atlantic (Table 2). Only one out of 41 haplotypes was common between the Atlantic and the Pacific. Diversity was significantly lower (1–6 haplotypes) for the other genera of the MCC. For all other genera within the Chrysogorgiidae except *Rhodaniridogorgia*, more haplotypes were found in the Indo-Pacific compared to the Atlantic. *Metallogorgia*, *Iridogorgia* and *Radicipes* each had one haplotype in common between the Atlantic and the Pacific (Table 2). Relative to the number of colonies sampled, *Metallogorgia* was the least variable within the MCC. Among 64 colonies, two *mtMutS* haplotypes were sampled. The first, corresponding to *Metallogorgia melanotrichos* Versluys, 1902, was found 62 times across the N. Atlantic, SW and NE Pacific. Although *M. melanotrichos* was previously thought to be restricted to seamounts, we sampled colonies on a continental slope (Bahama Escarpment). The other haplotype, corresponding to *Metallogorgia macrospina* Kükenthal, 1919, was found only twice, on the Norfolk and the Kermadec ridges (vicinity of New Caledonia and New Zealand, respectively). The type of this species was originally described from West Sumatra, consistent with our observations of a SW Pacific distribution.

**Table 2:** Bathymetric range and biodiversity within the monophyletic, deep-sea Chrysogorgiidae (MCC).

Genus	Data source	N.	Depth (m)	Total	Atl.	Ind.	Pac.	Ant.
*Chrysogorgia*	morphology	615 (1589)	31–4327	63	13	5	51	1
	*mtMutS* gene	101	101–3860	41	11	0	31	0
*Metallogorgia*	morphology	120 (159)	570–2262	4	2	0	3	0
	*mtMutS* gene	64	810–2262	2	1	0	2	0
*Iridogorgia*	morphology	40 (49)	567–2311	5	4	0	1	0
	*mtMutS* gene	25	752–2311	4	2	0	3	0
*Rhodaniridogorgia*	morphology	5 (6)	568–2229	2	1	0	1	0
	*mtMutS* gene	2	663–2229	2	1	0	1	0
*Radicipes*	morphology	78 (208)	196–3580	7	4	2	3	0
	*mtMutS* gene	16	308–3000	6	3	0	4	0
*Pseudochrysogorgia*	morphology	3 (5)	861–1429	1	0	0	1	0
	*mtMutS* gene	5	861–1429	1	0	0	1	0

Diversity: morphology estimate based on number of nominal species; genetic estimate based on number of *mtMutS* haplotypes. N: sample size (morphology: number of biogeographic records, and minimum number of colonies in parentheses), Atl: Atlantic Ocean, Ind: Indian Ocean, Pac: Pacific Ocean, Ant: Antarctic Ocean. Some depth estimates are based on depth ranges from trawling stations, in which case minimum and maximum depths were averaged (see notes in the Material and Methods section).

### Bathymetric and Geographic Distribution

A total of 985 biogeographic records (2345 coral colonies) were gathered from the literature, museum collections, and our own collecting. Of these, 24 records had a depth range considered too large relative to the average depth, and were removed from the dataset (see Methods). In addition, two records were removed as being extremely shallow, and most probably errors (*Metallogorgia* sp. USNM 56792 and *Iridogorgia pourtalesii* Verrill, 1883 Blake station 259; see [16] and [5]). The final database used in the analysis contains 959 records representing 2302 colonies.

**Figure 4:**
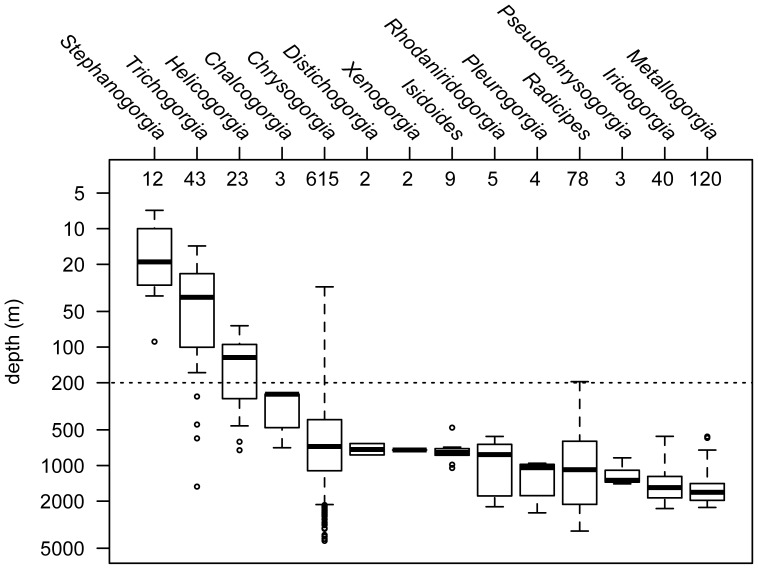
Depth distribution of the 14 Chrysogorgiidae genera based on 959 depth records. Depth records are summarized as box-and-whisker plots displaying the minimum, first quartile, median (bolded line), third quartile, and maximum values. Statistical outliers (>1.5x the inter-quartile range) are presented as open circles. Genera are sorted by increasing median depth (in meters, log scale) and sample size (number of biogeographic records) is provided on the top side of the plot. The 200 m isobath is represented (dashed line) as an arbitrary limit between deep and shallow waters.

The genera *Metallogorgia* and *Iridogorgia*, relatively well sampled (*n* = 120 and 40, respectively), are found exclusively in deep waters between 567 and 2311 m. The genera *Radicipes* and *Chrysogorgia*, well sampled as well (*n* = 78 and 615), are more wide ranging, and *Chrysogorgia* in particular appears as a depth generalist, ranging from 31 to 4327 m (both extremes correspond to unidentified *Chrysogorgia* specimens held at the NMNH, Smithsonian Institution). *Stephanogorgia* is the only shallow-water specialist (*n* = 12, range 7–37 m, with one outlier, USNM 79628, sampled at 90 m and identified by Frederick Bayer), and *Helicogorgia* and *Trichogorgia* (*n* = 23 and 43), while being found predominantly in waters shallower than 200 m, were occasionally reported from about 1000 m deep (Figure 4). Many genera are only known from a few specimens and estimating their depth distribution is therefore inherently biased. *Pseudochrysogorgia*, *Pleurogorgia*, *Isidoides*, *Xenogorgia*, *Distichogorgia* and *Chalcogorgia* are all known from eight specimens or less. All are found in waters below 200 m (n = 24, 250–2509 m), and most have a narrow depth distribution (Figure 4). The depth distributions of haplotypes and nominal species were very similar (Tables 2 and S1).

**Figure 5:**
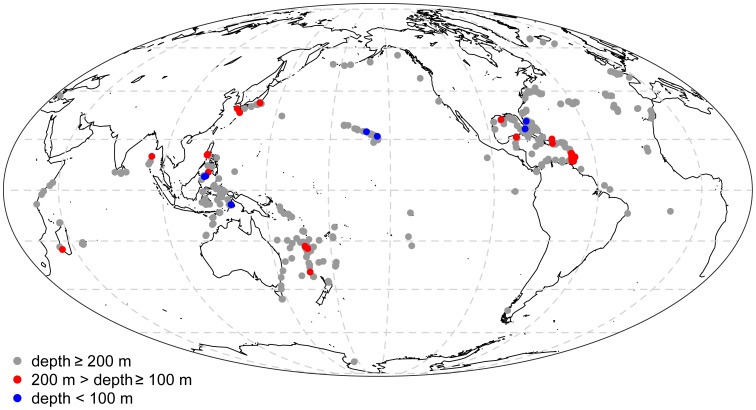
Geographic distribution of *Chrysogorgia* based on our biogeographic database (all 634 records, Mollweide projection). Records are displayed for three different depth ranges (in meters).

Among the genera belonging to the MCC, nine out of 89 species extend shallower than 200 m, all of them belonging to *Chrysogorgia*. Specimens reported in the taxonomic literature were collected from the NW Atlantic (*C. desbonni* Duchassaing & Michelotti, 1864, *C. thyrsiformis* Deichmann, 1936, *C. fewkesii* Verrill, 1883: [7], [8], [16], [17]), the NW Pacific (*C. sphaerica* Aurivillius, 1931, *C. dichotoma* Thomson & Henderson, 1906, *C. axillaris* (Wright & Studer, 1889), *C. geniculata* (Wright & Studer, 1889), *C. cupressa* (Wright & Studer, 1889): [18]–[21]), and the NW Indian Ocean (*C. dichotoma* Thomson & Henderson, 1906: [22]). With the exception of one specimen (*C. cupressa*, from the Banda Sea), all of these colonies were sampled in the northern hemisphere, south of 34°N (Figure 5). An additional species, held at the NMNH (USNM 91906) and identified by Charles Nutting as *C. curvata* Versluys, 1902 (but not included in his 1908 monograph [23]), was collected in the NE Pacific between 37–55 m depth. This specimen was confirmed by Dr. Stephen Cairns (Smithsonian Institution) as *Chrysogorgia*; this very shallow occurrence is therefore either real, or reflects a recording error of station data, or is the result of remnant specimens being retained in the net between dredges (Dr. Cairns notes that the preceding station for this collection was dredged from 1384–1829 m). One of these nine species, *C. dichotoma*, was exclusively found between 165 and 188 m, but is known from only three specimens collected in Japan and the Bay of Bengal [20], [22]. Other species extend much deeper, five of them being found below 1000 m.

**Figure 6:**
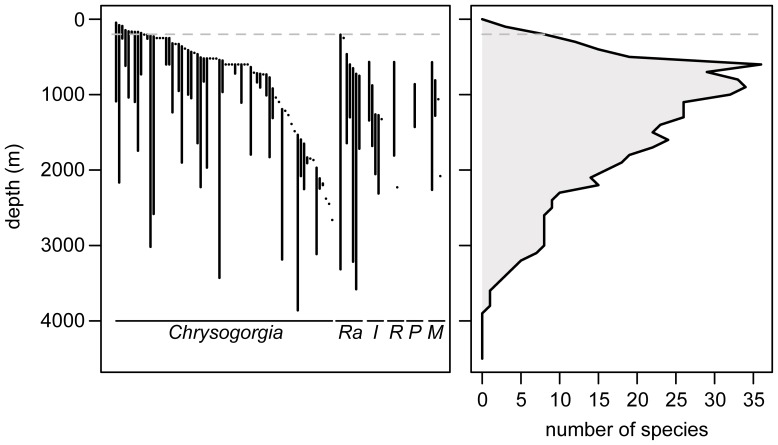
Depth range of 89 species (541 records) from the MCC (left) and resulting species diversity gradient (right). On the left panel, each segment links the maximum and minimum collection depths for a particular species. Species within genera are sorted by increasing median depth (m). The 200 m isobath is represented as a dashed line. Ra: *Radicipes*, I: *Iridogorgia*, R: *Rhodaniridogorgia*, P: *Pseudochrysogorgia*, M: *Metallogorgia*.

Among the genera belonging to the MCC, a diversity peak was observed at 600 m (36 species). Species diversity was maximum between 600 and 1000 m (29–36 species). All genera of the MCC were sampled within this depth interval. Diversity decreased progressively from 1000 m to 3860 m, the depth at which the last specimen identified to the species level was collected (Figure 6). This colony is a *Chrysogorgia* that was recently described by Pante and Watling [24], and was collected on Retriever Seamount in the NW Atlantic. Observations of a diversity gradient relative to depth are, of course, intimately linked to sampling effort in octocorals [25].

## Discussion

### Family Monophyly and the Diagnosis of the Chrysogorgiidae

Genetic data strongly suggest that the Chrysogorgiidae is polyphyletic as currently described. In our analysis of *mtMutS*, chrysogorgiid genera appeared in three different clades: the “monophyletic Chrysogorgiidae clade” (MCC), the *Pleurogorgia*/*Stephanogorgia*/*Trichogorgia*/Ifalukellidae clade, and the *Isidoides*/Keratoisidinae clade. The position of *Helicogorgia* was labile among phylogenetic trees, being either sister to the MCC, or sister to the clade composed of the Primnoidae and the MCC. The very limited data available for *Chalcogorgia* suggests a close affinity with *Helicogorgia*.

The polyphyly of the family calls for a re-assessment of the diagnostic characters used to differentiate the Chrysogorgiidae. The family is currently diagnosed as (e.g., [8]): The axis is unjointed, solid (non-spicular), and made of concentrically-layered scleroproteinaceous material; these layers are not undulated; the axis surface is smooth and has a metallic or iridescent sheen; colonies are branched or unbranched; the holdfast is strongly calcified, and discoidal or rhizoidal; polyps are contractile, not retractile, and are arranged in rows, sometimes bi- or multiserially, never in whorls or on opposite sides of the branch; sclerites are mostly flat scales, plates, rods and needles; when exposed to polarized light, scales show a distinct circular (not cruciform) extinction pattern (for an example see p. F221 in [26]).

*Trichogorgia* and *Stephanogorgia* have in common biserially-arranged polyps, which contrasts with the organization in rows found in genera of the MCC. This suggests that polyp arrangement as a diagnostic character of the Chrysogorgiidae should be revised. Three of the four genera for which genetic information are lacking are characterized by bi- or multiserially-arranged polyps: *Chalcogorgia*, *Distichogorgia* and *Helicogorgia*. If indeed polyp arrangement is diagnostic of phylogenetic placement, then we can predict that these three genera should not belong to the MCC. Similarly, two genera (*Pleurogorgia* and *Helicogorgia*) have ornamented sclerites that are uncharacteristic of the Chrysogorgiidae. As *Pleurogorgia* does not belong to the MCC, it can be predicted that *Helicogorgia* is probably not a true chrysogorgiid. Our *Pleurogorgia* specimens are small whips with polyp and sclerite morphology, and biogeography, consistent with published records for this genus [19], [23]. Both described species of *Pleurogorgia* are branching, however, and our specimens might belong to undescribed species (Alderslade, pers. com) or colonies at an early pre-branching life stage.

On the other hand, some characters currently used in the diagnosis might not be good indicators of the uniqueness of chrysgorgiids. Nutting [27] initially placed the genus *Isidoides* in the Gorgonellidae (now Ellisellidae). He noted its unusual morphology and the resemblance of sclerites to those found in some isidids. Bayer and Stefani [28] later noted that the fine, smooth scales of *Isidoides* are typical of the Chrysogorgiidae. Although exactly when the genus *Isidoides* was placed in the family Chrysogorgiidae is unclear, the genus appears in a key of the family as early as 1979 [29], but without justification. Later, Bayer and Grasshoff [30] stated that *Isidoides* should belong to the Chrysogorgiidae rather than the Ellisellidae. Our phylogenetic data clearly show that *Isidoides* does not belong to the Chrysogorgiidae, and supports Nutting’s initial suggestion that *Isidoides* might be related to bamboo corals.

There is no published record that all nine characters diagnostic of the family have been scored for all extant genera. For example, *Chrysogorgia* is the only genus for which clear evidence was provided that, when exposed to polarized light, scales show a distinct circular extinction pattern [26]. For this study, we confirmed that specimens from all genera of the MCC show a circular extinction pattern. However, it is also the case for *Pleurogorgia*, *Stephanogorgia*, *Helicogorgia*, *Xenogorgia*, *Isidoides*, and even bamboo corals. Circular light extinction pattern is therefore clearly not suited as a diagnostic feature of the MCC. Similarly, the composition and arrangement of axial layers are rarely reported. These results imply that characters diagnostic of the Chrysogorgiidae need to be re-evaluated for these genera; the diagnosis of the family will have to be revised.

### Trichogorgia, Stephanogorgia, Pleurogorgia, and the Ifalukellidae

Three of the 14 chrysogorgiid genera (*Trichogorgia*, *Stephanogorgia*, *Pleurogorgia*) formed a well-supported clade with the ifalukellid genera *Plumigorgia* and *Ifalukella*. The short genetic distance (*mtMutS*, uncorrected p: 0.72%) between the latter two genera supports the validity of the Ifalukellidae as a family (at *mtMutS Plumigorgia* and *Ifalukella* differ by only one amino acid, and have an identical indel structure, which is often quite variable among calcaxonians). Based on the taxa (Calcaxonia: Chrysogorgiidae, Primnoidae, Isididae) used in the study of McFadden et al. [15], the maximum intra-familial uncorrected p (at *mtMutS*) distance is 4.9%, while the minimum inter-familial distance is 3.8%. The distance between *Trichogorgia* and ifalukellids is <3.4%. Based on this criterion, we can legitimately suggest that *Trichogorgia* be considered as an ifalukellid. On the other hand, the genetic distances between *Stephanogorgia*, *Pleurogorgia*, and the *Trichogorgia*/Ifalukellidae group are all >6.7%, suggesting that two new families may need to be erected.

The phylogenetic grouping of *Trichogorgia*, *Stephanogorgia*, *Pleurogorgia* and the Ifalukellidae is only preliminary, and more comprehensive genetic and morphological analyses will have to be conducted. Only a single *mtMutS* haplotype could be used to represent each genus. More extensive taxon sampling will be required to comment further on the status of these genera, confirm their placement on the Calcaxonia tree, and rule-out spurious effects of long-branch attraction (e.g., [31]). There is nevertheless congruence among morphology, ecology and phylogeny. Morphologically, *Trichogorgia*, *Stephanogorgia*, and the Ifalukellidae share small, smooth sclerites in the form of scales and plates. In addition, both *Trichogorgia* and *Ifalukella* have relatively few sclerites in their tissues, to an extreme for *T. capensis* Kükenthal, 1919, which is completely lacking them. Also, the Chrysogorgiidae are defined as having an axis made of concentric layers that are not undulating. These layers are slightly undulating in the Ifalukellidae [26], [32]. However, the descriptions of *Stephanogorgia*, *Trichogorgia* and *Pleurogorgia* give no indication of how the layers are arranged. Examination of this character may inform us on the relationship of these taxa to ifalukellids. Ecologically, *Trichogorgia* and *Stephanogorgia* are two of the shallowest chrysogorgiid genera, and *Stephanogorgia* is a tropical lagoon-dwelling taxon. These observations are consistent with the fact that ifalukellids are strictly found above 50 m on coral reefs (e.g., [32], [33] and collection records from the Smithsonian Institution).

### Genus Monophyly and Genetic Diversity

With the exception of *Iridogorgia* and *Rhodaniridogorgia*, all chrysogorgiid genera were monophyletic. Specimens of *Iridogorgia* and *Rhodaniridogorgia* have been separated by the morphology of their spiral (*Iridogorgia*: main stem coiled; *Rhodaniridogorgia*: main stem wavy) and the morphology and placement of sclerites (sclerites are larger in *Rhodaniridogorgia*, and consistently present in the branch coenenchyme) [5]. In contrast, genetic distances separating these genera were consistent with intra-generic variation. These genera might have diverged too recently for our markers to have detected their reciprocal monophyly. Therefore, more specimens and a fine-scale genetic analysis will be needed to decide whether these genera should be synonymized.

Both *Radicipes* and *Chrysogorgia* were supported as monophyletic genera, although the relationship between them and the clade composed of *Metallogorgia*, *Pseudochrysogorgia*, *Iridogorgia* and *Rhodaniridogorgia*, was difficult to recover. As predicted in the taxonomic literature (in particular [19]), *Chrysogorgia* appeared as a highly diversified genus: 73% of haplotypes within the MCC (41 out of 56) belong to *Chrysogorgia*. Pante and France [14] showed that the distribution of intra-generic and inter-generic genetic distances between *Chrysogorgia* and *Radicipes* are greatly overlapping, with some *Chrysogorgia* haplotypes being more divergent than *Chrysogorgia*-*Radicipes* pairs. In contrast to the relatively high level of divergence within *Chrysogorgia*, the very short genetic distances between most *Chrysogorgia* clades suggest a rapid diversification of the genus.

The available evidence does not allow us to determine whether the diversification of *Chrysogorgia* is the result of an adaptive radiation. Theory predicts that an adaptive radiation would be associated with a high diversity of filled ecological niches. Four main hypotheses should be evaluated to test for adaptive radiation: (1) all taxa share a common ancestor, (2) there is a correlation between the environmental conditions and phenotypes, (3) new traits are adaptive, and (4) speciation is rapid (reviewed in [34]). Although the first requirement is met, other hypotheses remain to be tested.

First, ecological information associated with individual *Chrysogorgia* haplotypes is scarce; most haplotypes are singletons (i.e., sampled only once), and correspond to colonies sampled using trawls. In the case of trawled specimens, ecological information can be inferred from the whole catch (residual substrate and associated species). Specimens collected using underwater vehicles have the advantage of being associated with more extensive ecological data, but are a minority at the moment. While ecological data is scarce, some observations are congruent with an adaptive radiation. *Chrysogorgia* is the most widely-distributed chrysogorgiid genus, both geographically and bathymetrically (Table 2). Specimens are found from soft and hard substrates (e.g., [35]), and in continental and oceanic environments (e.g., [8], [36]). *Chrysogorgia* colonies are characterized by rhizoidal and discoidal holdfasts, consistent with life in soft and hard substrates.

Second, phylogenetic relationships among *Chrysogorgia* haplotypes require more than sequencing of the 5′ end of *mtMutS*. Our comparative analysis shows that the relationships of some *Chrysogorgia* haplotypes could be resolved using *mtMutS*, *cox1* and 18S together. Future research efforts will include a more comprehensive molecular analysis of *Chrysogorgia*, and the mapping of ecological and morphological characters on a better-resolved phylogeny. This exercise, however, might be complicated by the fact that morphological characters are notoriously plastic (e.g., [37]–[40]) and convergent [41], [42] in the Octocorallia. In addition, determining whether morphological traits confer an adaptive advantage will be technically very challenging.

Finally, studying the pace of speciation in the Octocorallia remains a daunting task, as informative markers that are variable at the intra- and inter-population levels are not yet readily available ([15] and references therein). Concepcion et al. [43] reported intra-specific variation at SRP54 (single-copy nuclear intron of the signal recognition particle 54) in the octocoral *Carijoa*, but preliminary results suggest this marker does not reveal more haplotypes than *mtMutS* in the Isididae (France, unpublished), and is very challenging to work with in the Chrysogorgiidae (Pante, unpublished).

### Relationships of Evolutionary History, Depth, and Biogeography

The MCC formed a well-supported monophyletic clade within the Calcaxonia, sister to the well-supported family Primnoidae and sub-family Keratoisidinae (Isididae). The Primnoidae (“the quintessential deepwater octocoral family,” [44]) and Keratoisidinae are largely deep-water taxa. The phylogenetic position of the MCC within the Calcaxonia, and the fact that it mostly comprises deep-water taxa (80/89 species found strictly below 200 m) support the hypothesis that the MCC diversified in the deep sea from a deep-sea ancestor.

*Metallogorgia*, *Iridogorgia*, *Rhodaniridogorgia* and *Pseudochrysogorgia* formed a strong monophyletic group within the MCC, and specimens within this clade were exclusively sampled between 663 and 2311 m depth (Table 2). Observations from our biogeographic database confirmed that members of this group were exclusively confined to deep waters (567–2311 m). In contrast, specimens from the *Radicipes* and *Chrysogorgia* clades had a significantly wider depth range, extending between 101 and 3860 m (biogeography database: 31–4327 m). There is therefore a strong dichotomy between narrow-ranging (depth specialists) and wide-ranging (depth generalists) taxa within the MCC. Of all genera, *Chrysogorgia* was by far the widest-ranging taxon, both geographically and bathymetrically, and genotyped specimens were found as shallow as 100.3–101.7 m (average 101 m, Northern Gulf of Mexico). Phylogenetically, shallower specimens are nested within a clade of deep-water taxa (the MCC); we can therefore hypothesize that the shallowest *Chrysogorgia* have evolved from deep-water ancestors.

Lindner et al. [45] found evidence of four separate emergence events (three tropical, one temperate) leading to the colonization of deep stylasterid hydrocoral lineages into shallow waters (<50 m). As in their study, we found that the shallowest *Chrysogorgia* specimens occurred at tropical and subtropical latitudes (5°S - 34°N). Faunal exchange between deep and shallow water may occur where biophysical barriers are permeable (e.g., [46]). Emergence of deep-sea species may therefore occur at high latitude, where vertical movement of species adapted to cold-water may be facilitated (e.g., [47]–[49]). We have no evidence of *Chrysogorgia* from shallow waters at high latitudes (shallowest colony >40° latitude: *C. flexilis*, 219 m depth, coast of Chile; [18]), and the only species known from Antarctica was sampled between 445 and 448 m (*C. antarctica* [2]). Polar regions remain under-sampled, and additional sampling at high latitudes may reveal the presence of *Chrysogorgia* in shallow, cold waters. However, sampling efforts (>350 octocorals sampled) by us and colleagues in the Aleutian Islands (51–53°N) recovered no chrysogorgiids shallower than 1357 m, despite many other octocorals collected between 25–190 m [50], [51]. Faunal exchange between deep and shallow waters may have occurred at lower latitudes when deep waters were warmer than at present, between the Mesozoic and the early Cenozoic (180–50 mya, [48], [49]). This coincides with the period of opening of the Tethys Sea, and is consistent with the tethyan distribution of the shallowest *Chrysogorgia* specimens. Unfortunately, there are no published records of chrysogorgiid fossils (and no records on the Paleobiology Database) to support this hypothesis. In addition, our shallowest *mtMutS* haplotype was found between 100.3 and 101.7 m, and genetic evidence for colonization of *Chrysogorgia* above 50 m depth (as suggested by our biogeographic database) is yet unavailable. Unfortunately, available historical material from shallow depths was collected between 1868 and 1976, and we have had limited success extracting PCR-amplifiable DNA from such material. Future efforts might therefore depend on the collection of fresh tissue.

## Materials and Methods

### Collections and DNA Extraction

Most specimens were collected during a series of deep-sea coral expeditions to Hawaii (1996, 2009), Alaska (2004), the North Atlantic (2003–2005), New Caledonia (2008), and the Bahama Escarpment (2009). Additional specimens from museum collections (via formal loan requests) and colleagues were utilized (Table S1; we confirm that the museums and colleagues who provided these specimens gave us permission to use them). The species represented here are not protected and do not require collecting permits. Sampling in the North Atlantic was mostly in international waters, and when inside of the US EEZ, was outside of National Marine Sanctuaries and fishery-closed areas focused on coral conservation; no permits were required. Specimens from Alaska were collected under State of Alaska Fish Resource Permits CF-04-009 and CF-06-013. Specimens from Hawaii were either collected outside of state waters, or in the Marine National Monument under permit PMNM-2009-053. Collecting in the Bahamas was done with permission from the Department of Marine Resources, Ministry of Agriculture and Marine Resources of The Bahamas, as assigned to the University of Miami/RSMAS. Collecting in New Caledonia was done within the French EEZ, by a French boat, and no permit was required. Whole colonies or portions thereof were sampled using remotely-operated vehicles (ROV), human-occupied vehicles or scientific trawls. Fragments for genetic analyses were preserved in 80–100% ethanol, RNAlater (Ambion), or frozen at −80°C. DNA was extracted using a modified CTAB protocol [10] or using the MasterPure DNA purification kit (Epicenter).

### DNA Amplification and Sequencing

Three gene regions were targeted. First, we attempted to PCR-amplify the 5′ end of the mitochondrial, protein-coding *mtMutS* for all available specimens. Although this gene has been most frequently referred to as *msh1* in the octocoral systematics literature, we will refer to it as *mtMutS* throughout this paper as this term makes fewer assumptions about its evolutionary origins [52]. *mtMutS* was chosen because it is one of the most variable and informative markers available for octocorals to date (16S: [10]; *mtMutS*, *nd3*, *nd4l*: [53]; *cox1*: [54]; [55]; *nd2*, *nd3*, *nd6*: [56]; *mtMutS*, *cox2*-IGR-*cox1*: [15]; *mtMutS*, *cox1*, *nd2*, *nd3*, 16S, 28S, ITS2: [57]). All chrysogorgiids and outgroup taxa, except the Keratoisidinae, were amplified by priming in the flanking gene *nd4l* with upstream primer ND4L2475F [58] and extending into *mtMutS* with downstream primer MUT3458R [59]. This last primer was later replaced by MUTChry3458R (Table 3), a novel primer that is a perfect match to primnoids, isidids and chrysogorgiids tested to date. Mitochondrial gene order is not conserved across the sub-order Calcaxonia [58]. In the isidid sub-family Keratoisidinae, *mtMutS* is flanked by *cox3* at its 5′ end. Thus, primers CO3BAM5647F and MUT3458R were paired to amplify *mtMutS* in this group of taxa. Internal primers were used when DNA was degraded (primers 1–16, Table 3).

**Table 3:** PCR primers used in the present study to amplify targeted gene regions.

	Name	Sequence (5′ ––3′)	Gene	Product size	Cycle	Reference
1	CO3Bam5657f	gctgctagttggtattggcat	*cox3*	1–15: 1014 bp	94:20,55:30,72:50	[58]
2	ND4L2475F	tagttttactggcctctac	*nad4L*			[58]
3	ND42625F	tacgtggyacaattgctg	*nad4L*	3–10: 476 bp	94:20,50:30,72:30	[58]
4	MSH2714F	cttaatggaggagaattattc	*mtMutS*			this publication
5	MSH2806F	taactcagcttgagagtatgc	*mtMutS*			[58]
6	msh2864r	gaggcaacttgttcaatgggaggtg	*mtMutS*			this publication
7	MSH3010F	ggataaaggttggactattatag	*mtMutS*	7–16: 448 bp	94:20,55:30,72:30	[6]
8	MSHLA3034R	cctgagatactgcgcgttgtttaggccccg	*mtMutS*			[58]
9	MSH3055R	ggagaataaacctgagayac	*mtMutS*			[58]
10	MSH3101R	gatatcacataagataattccg	*mtMutS*			[59]
11	MSH3186F	gccatgartgggcatagtata	*mtMutS*			this publication
12	msh3208r	atcgagcyactttgtccckgtc	*mtMutS*			this publication
13	MSH3332F	cttattaattggttggaa	*mtMutS*			this publication
14	MSH3350F	gccatgartgggcatagtata	*mtMutS*			this publication
15	MUT3458R	tsgagcaaaagccactcc	*mtMutS*	2–15: 940 bp	94:20,50:30,72:50	[59]
16	MUTChry3458R	tgaagyaaaagccactcc	*mtMutS*	2–16: 940 bp	94:20,50:30,72:50	this publication
17	MSH3841F	ctgcgttatgaggagattgckac	*mtMutS*			this publication
18	MSH4094F	cagtcggacctcaattagaatcg	*mtMutS*			this publication
19	MSH4332R	gaaggcataaccctccttactg	*mtMutS*	14–19: 920 bp	94:20,50:30,72:50	this publication
20	MSH4757R	gacttgcccgcaccatttactg	*mtMutS*			this publication
21	MSH4759F	tgtagctcatgatattag	*mtMutS*			[41]
22	MSH4915R	cgacctcaaaagtaccttgacc	*mtMutS*	18–22: 830 bp	94:20,50:30,72:50	this publication
23	MSH5065F	gcaacaattgaaagattraca	*mtMutS*			this publication
24	MSH5075R	gagtagamagarcgaaactag	*mtMutS*			this publication
25	MSH5376R	agctccacatatttcacac	*mtMutS*			this publication
26	16S5PR	tcacgtccttaccgatag	16S	21–26: 900 bp		[41]
27	COII8068xF	ccataacaggrctwgcagcatc	*cox2*			[15]
28	COIoctR	atcatagcatagaccatacc	*cox1*	27–28: 1080 bp	94:20,50:30,72:60	[54]
29	18S-Af	aacctggttgatcctgccagt	18S			mod. [76]
30	18S-Lr	ccaactacgagctttttaactg	18S	29–30: 620 bp	94:20,60:30,72:40	[77]
31	18S-Cf	cggtaattccagctccaatag	18S			[77]
32	18S-Yr	cagacaaatcgctccaccaac	18S	31–32: 710 bp	94:20,60:30,72:40	[77]
33	18S-Of	aagggcaccaccaggagtggag	18S			[77]
34	18S-Br	tgatccttccgcaggttcacct	18S	33–34: 620 bp	94:20,60:30,72:40	mod. [76]

Predicted fragment sizes (approximate, in bp) and PCR cycle profiles (temperature in °C: time in seconds) are given for the most commonly used primer pairs. Primer combinations are listed in the product size column, prior to the predicted fragment size, using the primer numbers defined in the first column. (mod.: modified from). Between 30 and 45 cycles were used for PCR.

For a set of taxa, additional gene sampling was performed. These taxa were chosen based on their position on the *mtMutS* phylogeny to achieve two goals: (1) to assess if additional sequencing would retrieve more variation from clades/taxa that show little or no variation at *mtMutS* (e.g., *Metallogorgia melanotrichos*), and (2) to further resolve phylogenetic relationships among the Chrysogorgiidae (e.g., relationship between *Radicipes* and *Chrysogorgia*). For one set (*n* = 64), *cox2*-IGR-*cox1* and 18S were amplified (primers 27–28 and 29–34). Among these (*n* = 42), the nearly complete *mtMutS* was amplified (2889–2997 bp; primers 1–26).

PCR was performed in 25 µL total volume using 1x TaKaRa *Ex Taq* buffer (Mg^2+^-free, proprietary composition), 1.5 mM of MgCl_2_, 0.4 mM of dNTP mix, 0.5 U of *Ex Taq* polymerase (TaKaRa Bio USA Inc., now Clontech), 0.24 µM of each primer (Operon Biotechnologies, Inc., now Eurofins MWG Operon), and 40 ng of DNA template (quantified using BioRad VersaFluor fluorometer and/or Thermo Scientific Nanodrop ND-1000 spectrophotometer). 2.5 µg of acetylated BSA (Promega) was added for problematic samples. PCR amplification conditions were optimized for each primer pair (Table 3 provides cycling profiles for the most commonly-used pairs). PCR products were purified either by excising bands from low-melting point agarose gels followed by an agarase digestion (5 U; Sigma-Aldrich Co.; [54]) or by an Exo-SAP digestion (2 U of ExoI and 0.2 U of SAP/1 µL of DNA; Fermentas; [60]). The majority of purified PCR reactions were cycle-sequenced using the ABI BigDye Terminator v1.1 Cycle Sequencing Kit (1/4 reactions) and purified using either an EtOH/EDTA precipitation or Sephadex G-50 columns (Sigma-Aldrich). Purified products were electrophoresed on an ABI PRISM (R) 3100 or 3130xl Genetic Analyzer. A fraction of the PCR products were purified with AmPure XP beads, cycle-sequenced using the ABI BigDye Terminator v3.1 Cycle Sequencing Kit (1/32 reactions) and purified using an EtOH/Sodium Acetate precipitation. These products were electrophoresed on an ABI PRISM (R) 3730xl Genetic Analyzer. All sequence traces were edited using Sequencher (TM) v4.7 (Gene Codes). DNA sequences of specimens representing each haplotype, for each biogeographic region, were submitted to GenBank (Table S1).

### Data Analysis

*mtMutS* sequences were translated to amino acids (Mold-Protozoan mitochondrial code) and aligned using MAFFT 6 (L-INS-i method, [61], [62]). TranslatorX was used to align nucleotides based on the amino-acid alignment [63]. There were no indels in the *cox1* alignment. The 18S dataset contained only single-nucleotide indels, and could therefore be aligned by eye. Saturation plots and transition/transversion ratios were computed for each dataset using the ape 2.5–2 package [64] in R 2.12 [65]. The effect of complex indel motifs at *mtMutS* (particularly the 5′ region) on phylogenetic reconstruction was investigated by constructing trees with and without indels. Gblocks 0.91b [66], [67] was used to eliminate indel regions that are poorly conserved, while keeping the regions that contain informative sites. All alignments are available from the authors upon request. The phylogenetic information content of all gene regions (5′ end of *mtMutS*, whole *mtMutS*, partial *cox1* and 18S) and these regions in combination was evaluated by calculating the number of variable and parsimony-informative sites in MEGA 5 [68] and constructing maximum-likelihood (ML) trees using PhyML 3.0 [69]. Node support was evaluated with 1000 bootstrap replicates (500 replicates for *mtMutS*, *cox1* and 18S concatenated). The most-likely model of evolution was inferred using jModelTest 0.1.1 [70], and PhyML was parametrized accordingly. MrBayes 3.1 [71], [72] was used on the CIPRES Portal [73] to produce phylogenetic hypotheses based on Bayesian statistics (6 nucleotide substitution types, 4×4 substitution model with I+G among-site rate variation; 5 million generations, 2 runs, 4 chains, sampling every 1000 generations, burnin of 25% equaling 1250 samples). Convergence was assessed by checking that (1) standard deviations of split frequencies were <0.007, (2) potential scale reduction factors were close to one (they varied between 1 and 1.06), and (3) plots of log likelihood values did not show visible trends over time. Trees were rooted to the Pennatulacea (sea pens), which are the non-calcaxonian octocorals that are the most closely related to the Calcaxonia, based on the results of [9].

### Biogeography Database

Biogeographic data were compiled from the taxonomic literature (37 published papers, one in press, one in preparation), collection information from museums (the Australian Museum (Sydney), the Florida Museum of Natural History (University of Florida, Gainesville), the Harvard Museum of Comparative Zoology, the Museum and Art Gallery of the Northern Territory (Darwin, Australia), the Muséum national d’Histoire naturelle (Paris, France), the National Institute of Water and Atmospheric Research (Wellington, New Zealand), the National Museum of Natural History (Smithsonian Institution), and the Yale Peabody Museum of Natural History (Yale University)), and our collections (including specimens provided by colleagues). Duplicate records (i.e., fragments from individual colonies held in multiple museums, use of material in different manuscripts) were removed. Depth distributions were compiled using sampling station information. Thirty five percent of the depth records (341 of 975) come from dredging or trawling, for which the minimum and maximum depth were reported. The average depth was computed for these records. However, in some cases the depth range was so large that it made the average meaningless. All records for which the depth range was more than half of the average depth were excluded from the dataset. A species-diversity profile across depth was computed for species belonging to the genera forming a monophyletic clade by counting the number of species found every 100 m between 0 and 4500 m. The biogeographic database is available from the authors upon request.

## Supporting Information

Figure S1 **Bayesian 50% majority rule consensus trees based on different markers (** ***mtMutS*** **,** ***cox1*** **and 18S) and marker combinations.** For *mtMutS*, the effect of indels on phylogeny inference was tested by removing them with Gblocks. Chrysogorgiidae taxa are either color coded (MCC) or have bolded branches (nonMCC).All trees are rooted to the Pennatulacea, except trees using the entire *mtMutS* gene (rooted to the Ellisellidae).
(PDF)

Table S1 **Collection date, geographic coordinates, depth, and genetic markers sequenced for specimens used in this study.** GenBank accession numbers representing each haplotype and biogeographic region are given in the final 3 columns.
(XLS)
