# Supplementary figures and images for: Deep-Sea Origin and In-Situ Diversification of Chrysogorgiid Octocorals
Source: PLoS One. 2012 Jun 18;7(6):e38357. doi: 10.1371/journal.pone.0038357 (PMC3377635; doi:10.1371/journal.pone.0038357)

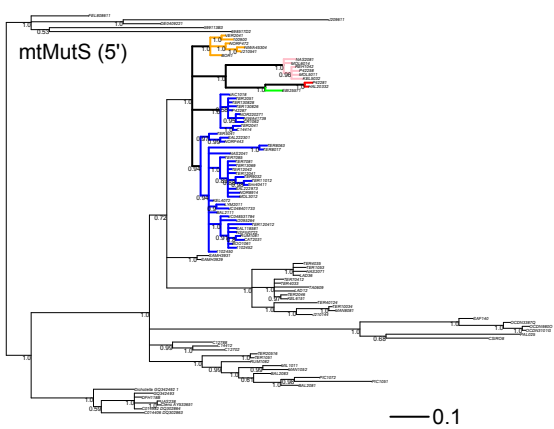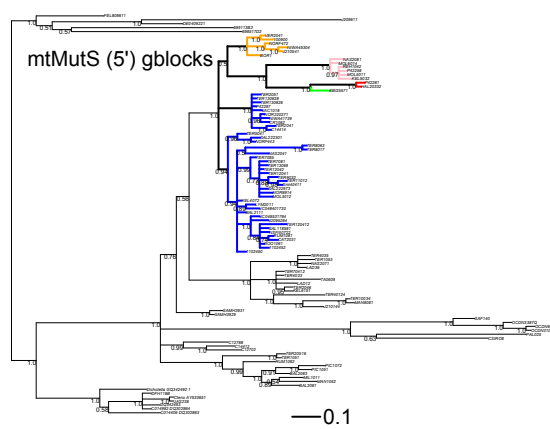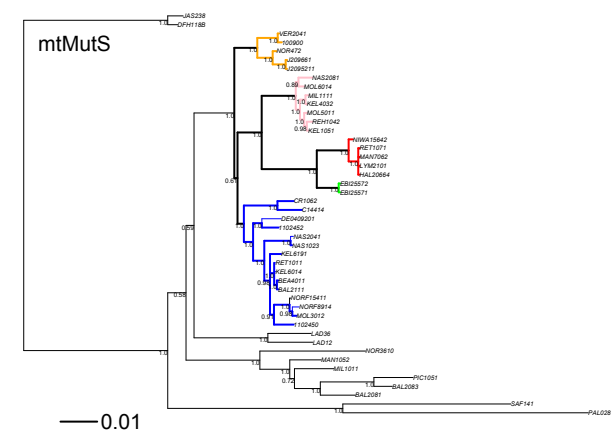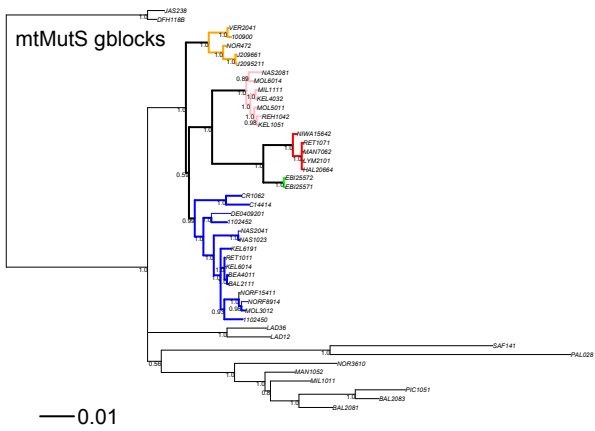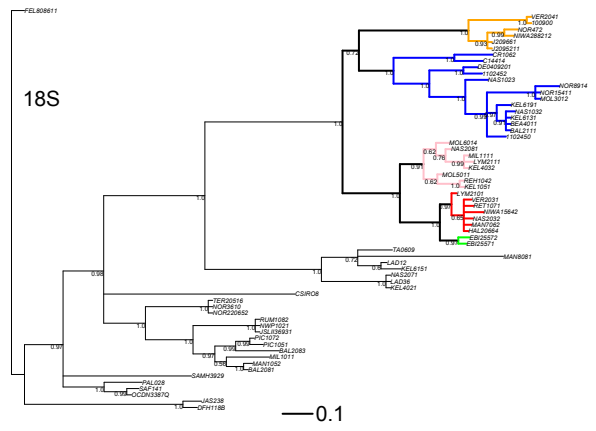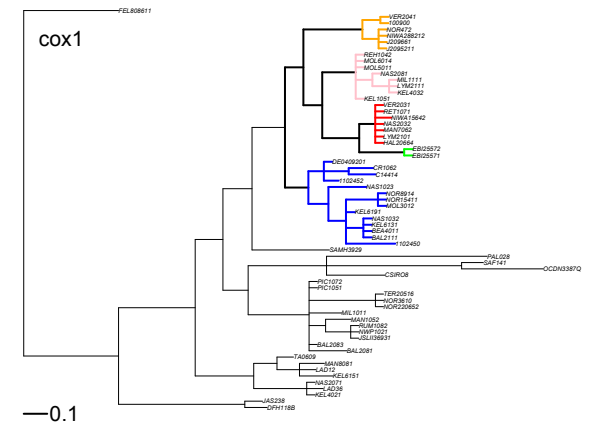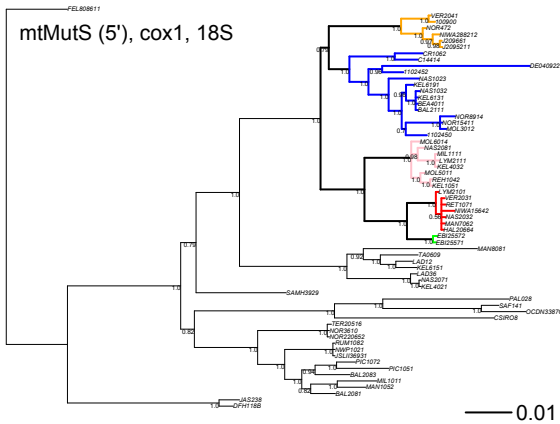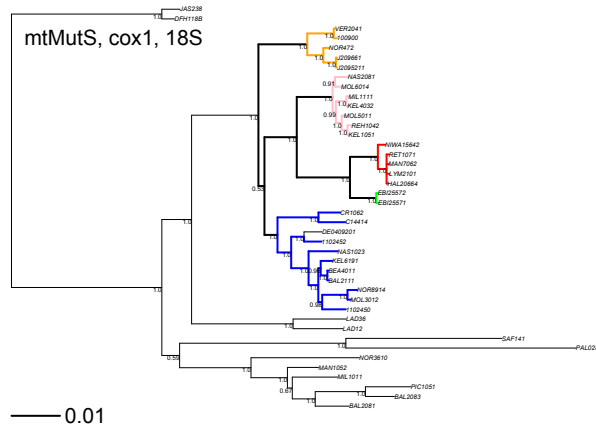

Supplement: Figure S1 — Bayesian 50% majority rule consensus trees based on different markers ( mtMutS , cox1 and 18S) and marker combinations. For mtMutS, the effect of indels on phylogeny inference was tested by removing them with Gblocks. Chrysogorgiidae taxa are either color coded (MCC) or have bolded branches (nonMCC).All trees are rooted to the Pennatulacea, except trees using the entire mtMutS gene (rooted to the Ellisellidae). (PDF) [file pone.0038357.s001.pdf]
